# Primary Burkitt Lymphoma of the Chest Wall

**DOI:** 10.1155/2012/746098

**Published:** 2012-03-26

**Authors:** Rommel Lu

**Affiliations:** Department of Pathology & Laboratory Medicine and Department of Medicine, Division of Hematology-Oncology, University of North Carolina at Chapel Hill, CB # 7600, Chapel Hill, NC 27599, USA

## Abstract

Burkitt lymphoma (BL) originating in the skin and soft tissue at any site is exceedingly rare. This paper is about a case of primary sporadic BL that presented as an isolated, rapidly enlarging chest wall mass arising from skin and/or soft tissue in an adult. As with other BL presentations, this patient was treated with aggressive chemotherapy with central nervous system (CNS) chemoprophylaxis, but he later died because of sepsis.

## 1. Introduction

Chest wall tumors are a heterogeneous group of benign and malignant disorders. Burkitt lymphoma (BL) originating in the skin and soft tissue at any site is exceedingly rare. This paper illustrates a case of primary sporadic BL that presented as an isolated, rapidly enlarging chest wall mass arising from skin and/or soft tissue in an adult and provides an overview of the clinicopathologic features of skin and soft tissue lymphomas including BL. 

## 2. Case Report

A 33-year-old previously healthy man presented with one-month history of a painful red nodule over his left chest wall. He initially noticed a discomfort in the area after chopping wood and later found a small “pimple-like” nonpustular nodule in the same area. After a week, the lesion became larger, erythematous, and painful. At an outside hospital, he underwent an incision and drainage of the chest wall mass without histological examination for a presumed diagnosis of chest wall abscess. Several days after drainage, a similar nodule recurred at the incised area, which rapidly grew in size. He also reportedly started experiencing fever and chills and was readmitted to the same hospital for a diagnosis of necrotizing chest wall infection. A debridement with excisional biopsy of the chest wall revealed atypical lymphoid cells, prompting transfer to our institution.

Upon transfer, a large, gaping, erythematous, and indurated wound with indistinct, thickened borders and extensive edema and necrosis of subcutaneous tissue and musculature of almost the entire left chest wall was noted. No palpable peripheral lymphadenopathy or organomegaly was observed. The serum lactate dehydrogenase (LDH) and uric acid levels at baseline were elevated at 1113 U/L (reference range: 336–610) and 9.2 mg/dL (reference range: 3.5–7.5), respectively, while his renal function was normal. The complete blood count (CBC) was significant for mild normocytic, normochromic anemia with hemoglobin of 11.0 g/dL.

**Figure 1:**
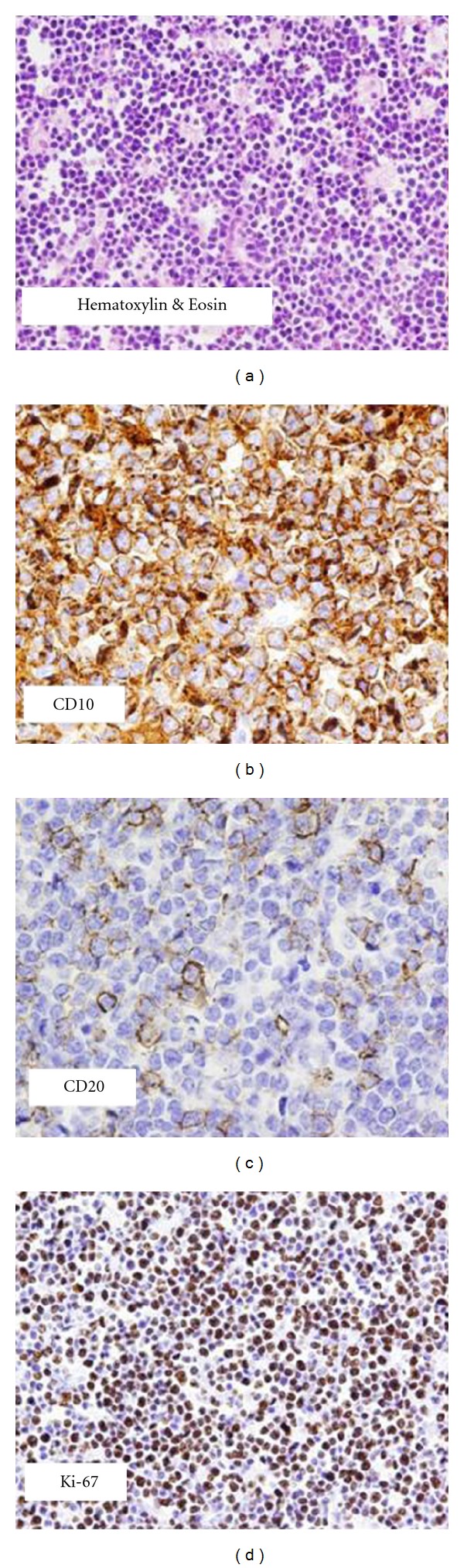
Slides revealed lymphocytes with deeply basophilic cytoplasm in a “starry-sky” pattern (a). The lymphoid population was CD10 (b), CD20 (c), and nearly 100% Ki-67 (d) positive.

**Figure 2:**
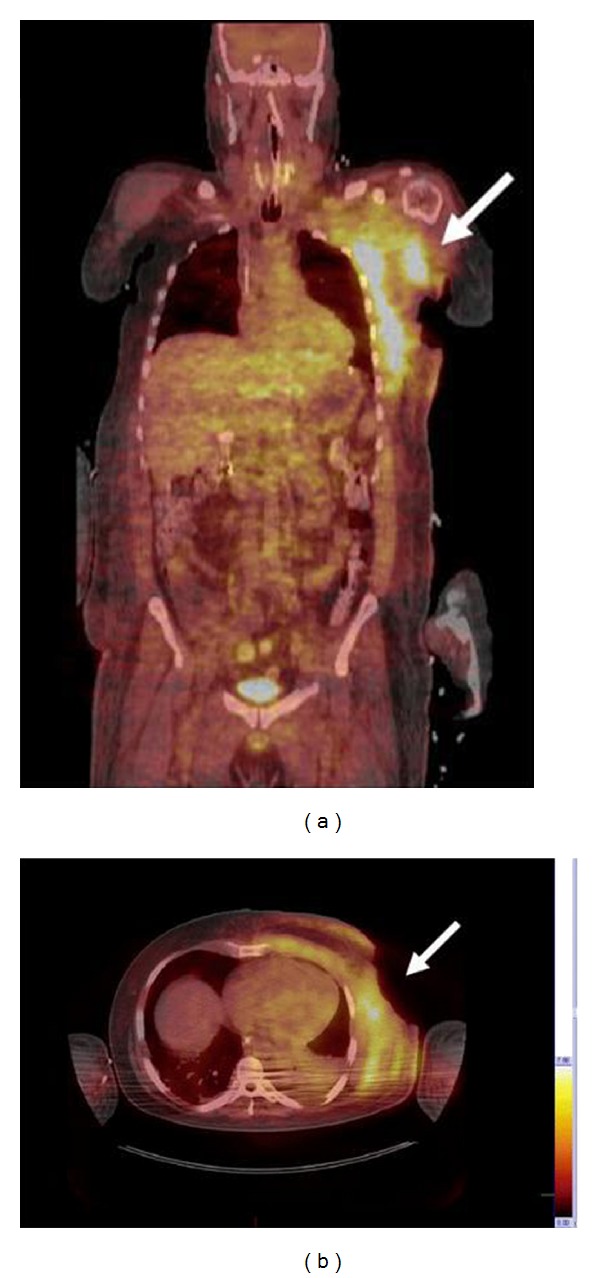
Staging PET scan showed a large subcutaneous defect of the left hemithorax (arrowheads). No additional involved sites were seen.

**Figure 3:**
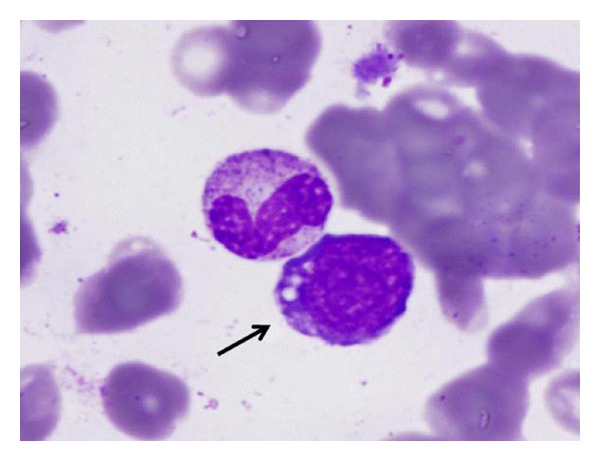
A classic BL cell seen in the bone marrow aspirate specimen (arrowhead).

In our institution, he underwent debridement and partial excision of the chest wall wound with biopsy. The histopathology revealed atypical lymphocytes with prominent nucleoli and deeply basophilic cytoplasm with abundant vacuoles. The lymphoid population was kappa light chain restricted, CD20 and CD10 positive, but negative for CD5 and Bcl-2. Nearly 100% were Ki-67 positive (Figure 1). Fluorescent in situ hybridization (FISH) revealed fusion signals with IgH/MYC t(8; 14) dual fusion probe, supporting the diagnosis of BL. Staging positron emission tomography/computerized tomography (PET/CT) scan demonstrated a large subcutaneous defect of the left hemithorax involving the dermis, subcutaneous tissue, and pectoral musculature, measuring 19.3 × 13.9 × 31.0 cm, with elevated [^18^F] fluorodeoxyglucose (FDG) uptake throughout the mass and maximal standardized uptake value (SUV) of 9.8 and an average of 6.2 (Figures 2(a) and 2(b)). No additional involved sites were identified including breast, mediastinum, axilla, pleura, and rib cage. The bone marrow aspiration and biopsy showed a few scattered BL cells (Figure 3) and hybridization pattern for IgH/MYC t(8; 14). The peripheral blood and cerebrospinal fluid showed no evidence of BL involvement. Serological tests for human immunodeficiency virus (HIV), hepatitis B and C were negative.

Three days after surgery, chemotherapy was initiated for high-risk BL. He received CODOX-M (Cyclophosphamide, doxorubicin, vincristine, cytarabine, and methotrexate) as cycle 1, followed by IVAC (Ifosfamide, etoposide, and cytarabine) with rituximab as cycle 2. He developed tumor lysis syndrome with cycle 1 but maintained normal end-organ functions. The chest wall edema and mass decreased, while the serum LDH normalized. Hydrogel wound dressing was applied over the left chest wall, and maggot debridement therapy was utilized after cycle 1 that resulted in a good granulation effect. Cycle 2 subsequently followed. However, five days after completion of cycle 2, and while receiving granulocyte colony-stimulating factor (G-CSF) support, he developed neutropenic fever with pneumonia which led to respiratory failure and he later died of septic shock. Blood cultures identified *Enterococcus faecium*. No autopsy was performed.

## 3. Discussion

Primary chest wall tumors are uncommon. Approximately 50% are malignant, and chest wall lymphoma accounts for less than 2% with extranodal diffuse large B-cell lymphomas being the most common subtype [1]. Direct extension from anterior mediastinal or axillary nodal disease is the most common type of spread to the chest wall, but occasionally, a chest wall mass due to lymphoma may arise from skin or soft tissue in the absence of mediastinal or axillary nodal or other extranodal involvement [2, 3]. Sporadic BL typically presents as an intraabdominal process and may involve the kidney, pancreas, liver, spleen, or ovary [4]. This paper is a case of primary skin or soft tissue involvement of the chest wall in the absence of apparent nodal mass or visceral disease as the initial manifestation of BL. While there are isolated reports of skin and soft tissue involvement by BL, these cases were in the setting of immunodeficiency state and were felt to be the result of either iatrogenic tumor seeding after nodal biopsies [5, 6], for which there is limited prospective evidence of such a mechanism, or local spread from intrathoracic lymph nodes in the setting of recurrent disease [6, 7]. Cases of BL arising in soft tissue of the lower extremity and chest wall during relapse in a child have also been reported [8, 9]. These reports suggest that hematogenous or lymphatic dissemination of malignant cells may be the mechanism underlying the development of skin and soft tissue involvement. Whether this case represents a BL that began in the skin and soft tissue and spread to the bone marrow, or began in the bone marrow and disseminated to the chest wall cannot be determined, but the minimal marrow involvement by BL would favor, to some extent, spread from the chest wall to the bone marrow. Furthermore, the absence of disseminated disease by PET/CT scan suggests that the skin and/or soft tissue of the chest wall were the site/s of origin in this case.

This patient's clinical presentation of an isolated, rapidly enlarging chest wall mass that developed despite surgical debridements, reflects the short doubling time of BL cells. BL is one of the most rapidly proliferating neoplasms known with a doubling time of as short as 25 hours [4]. It is a highly aggressive non-Hodgkin lymphoma (NHL) that is composed of monomorphic medium-sized B cells with numerous mitotic figures. The tumor cells express a mature B-cell profile, positive for CD19, 20, 22, and 79a as well as CD10 and Bcl-6 but negative for CD5 and Bcl-2. The genetic hallmark of BL is overexpression of c-MYC which is important in its pathogenesis and most commonly results from t(8; 14). The high mitotic rate and apoptotic tumor cell death associated with BL result in numerous tissue macrophages with ingested apoptotic tumor cells, creating the characteristic “starry-sky” appearance. These histopathologic features together with the c-MYC overexpression are diagnostic of BL. Categorically, BL is classified into endemic, sporadic, and immunodeficiency variants [10]. Therapeutically, BL is potentially curable with aggressive multiagent chemotherapy and central nervous system (CNS) chemoprophylaxis. In adults, treatment is adapted from pediatric regimens such as the French LMB regimens [11], German Berlin-Frankfurt-Munster (BFM) protocols [12], CODOX-M/IVAC [13], Stanford regimen [14], hyper-CVAD [15], and CALGB 9251 [16]. Evidence supporting the efficacy of an individual regimen over another is lacking. The CHOP (Cyclophosphamide, doxorubicin, vincristine, and prednisone) regimen used in NHL results in frequent relapses and is considered inadequate therapy [17]. The role of rituximab as part of BL treatment is yet to be defined as there are limited data incorporating rituximab into the various regimens [18], but it is certainly a reasonable addition based on the success of rituximab in other CD20-expressing NHLs. Bone marrow and CNS involvement are reported in 30–38% and 13–17% of adults with BL, respectively. In the absence of CNS involvement, CNS chemoprophylaxis is very important. Without prophylaxis, 30–50% of patients will relapse compared to 6–11% who received prophylaxis [19]. With aggressive chemotherapy, complete remission rates are 75–90% and overall long-term survival rate reaches 50–70% in adults [4]. In most published trials, the most commonly encountered toxicities were myelosuppression and infection, which unfortunately, were responsible for this patient's death.

In conclusion, this paper is about a case of *de novo* or primary BL arising in skin or soft tissue of the chest wall in an adult. Although no specific data exist regarding treatment approach, it is reasonable as with other BL presentations for these patients to receive aggressive chemotherapy with CNS chemoprophylaxis. The role of tumor debulking surgery is uncertain.
